# Antimicrobial Resistance in Slaughterhouses, Kenya

**DOI:** 10.3201/eid2910.230017

**Published:** 2023-10

**Authors:** Katie A. Hamilton, Sam M. Njoroge, Kelvin Momanyi, Maurice K. Murungi, Christian O. Odinga, Nicholas Bor, Allan F. Ogendo, Josiah Odaba, Joseph G. Ogola, Eric M. Fèvre, Laura C. Falzon

**Affiliations:** University of Liverpool, Liverpool, UK (K.A. Hamilton, E.M. Fèvre, L.C. Falzon);; International Livestock Research Institute, Nairobi, Kenya (K.A. Hamilton, S.M. Njoroge, K. Momanyi, M.K. Murungi, C.O. Odinga, N. Bor, A.F. Ogendo, J. Odaba, L.C. Falzon);; Kenya Medical Research Institute, Nairobi (S.M. Njoroge);; County Government of Busia, Busia, Kenya (A.F. Ogendo);; University of Nairobi, Kenya (J.G. Ogola);; University of Helsinki, Helsinki, Finland (J.G. Ogola);; County Government of Bungoma, Bungoma, Kenya (J.G. Ogola)

**Keywords:** antimicrobial resistance, bacteria, food safety, zoonoses, focus group discussion, stakeholders, antibiotics, integrated surveillance, Kenya

## Abstract

Slaughterhouses are hotspots for the transmission of antimicrobial-resistant pathogens. We conducted stakeholder discussions on antimicrobial-resistant pathogens within the slaughterhouse setting. Butchers were described as powerful stakeholders; challenges included limited funding and staff, inadequate infrastructure, and limited laboratory capacity. Slaughterhouse workers understood that their work increased their risk for exposure.

Antimicrobial resistance (AMR) in bacteria is one of the most serious global health threats of this century (*1*). Slaughterhouses act as disease hotspots because of frequent interactions between humans and animals (*2*), particularly in rural areas, such as western Kenya (*3*). Recent work showed that slaughterhouse workers are exposed to several zoonotic pathogens (*4*–*6*). However, little is known about the workplace risk for exposure to antimicrobial-resistant bacteria within the slaughterhouse context and the implications of workplace AMR exposure for public health and food safety in the region. This study engaged stakeholders in discussions on AMR within the slaughterhouse setting, with the objective of using baseline information to develop contextually relevant educational material and inform future research and improvements in work conditions.

## The Study

This work was embedded within a larger surveillance study conducted in Busia, Bungoma, and Kakamega counties in western Kenya (*7*). In total, we conducted 6 stakeholder discussions exploring AMR in the slaughterhouse context. First, we held focus group discussions (3–9 participants) with the county veterinary officers, subcounty veterinary officers, and meat inspectors in each of the 3 counties. Focus group discussions lasted 2–3 hours, during which other topics not reported in this study (i.e., legislation and animal welfare) were also discussed. Next, we held 3 workshops for slaughterhouse workers from Bungoma (n = 60), Kakamega (n = 90), and Busia (n = 40). Participants were distributed into groups, with a maximum of 20 participants per group, and discussions lasted 45–60 minutes.

**Figure 1:**
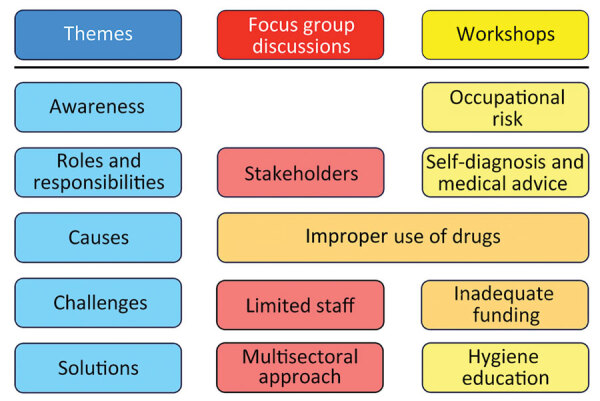
Themes identified from focus groups and in workshops in study of antimicrobial resistance in slaughterhouses, Kenya. Focus group discussions included county veterinary officers, subcounty veterinary officers, and meat inspectors; workshops included slaughterhouse workers. Blue indicates overall themes; red indicates results from focus group participants; yellow indicates results from workshop participants; orange indicates answers shared by both groups of participants.

The purpose and modus operandi of the discussions were explained, and written consent was obtained from each participant. Discussions were held in English or Kiswahili, as required, and were led by moderators who used an interview guide with broad, open-ended questions (Appendix 1). All discussions were recorded and later transcribed verbatim, and translated from Kiswahili to English as needed, by an investigator. Two investigators (K.A.H., L.C.F.) thematically analyzed all transcripts, and inductively derived findings by using an interpretive–descriptive approach to identify themes emerging from the data (*8*). Each investigator coded all transcripts separately, before discussion and consensus on the main themes (Figure 1; Appendix 2).

**Figure 2:**
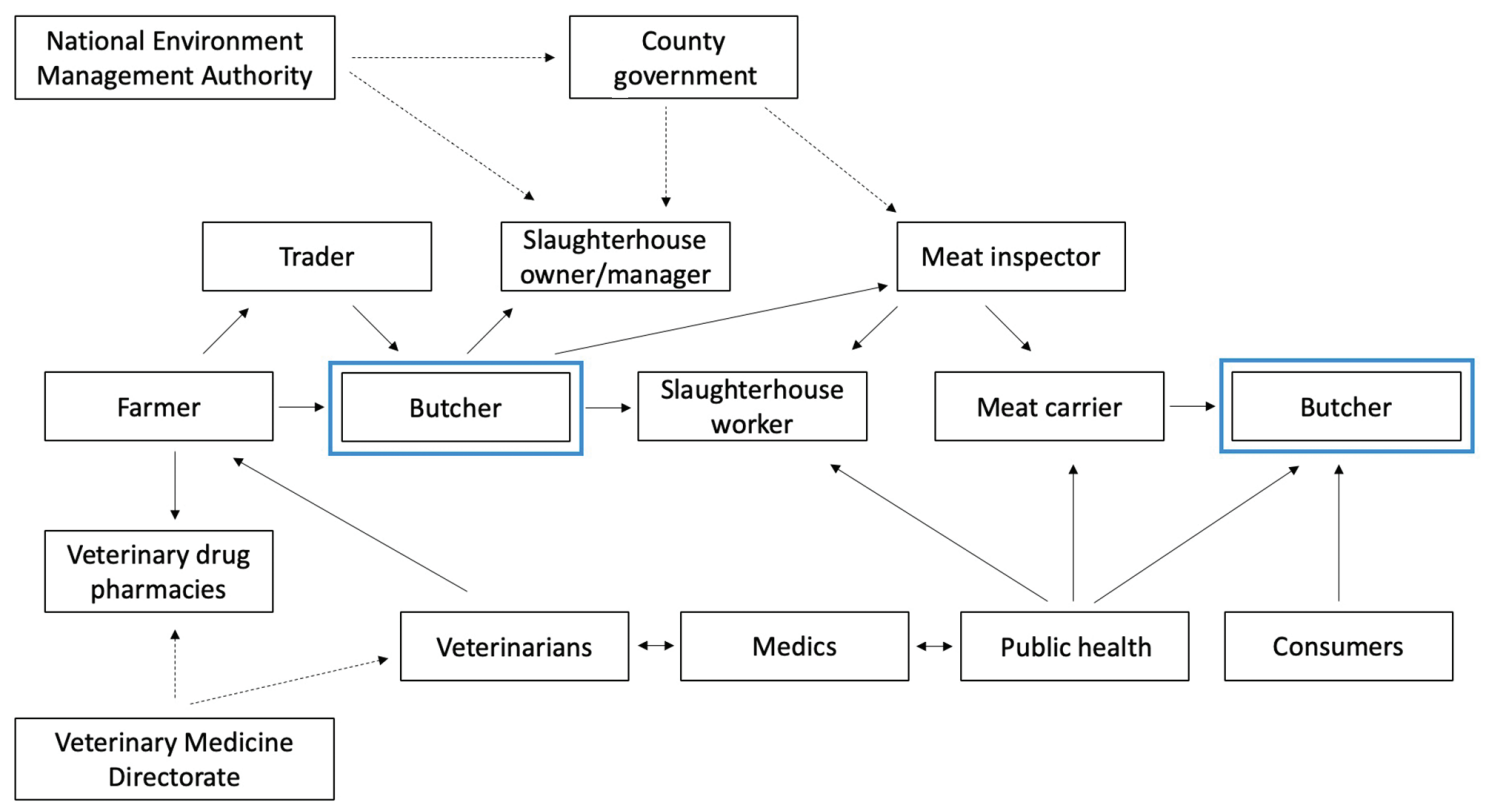
Relationship between stakeholders in study of antimicrobial resistance in slaughterhouses, Kenya. The chart shows relationships within the slaughterhouse context identified in focus group discussions conducted with county veterinary officers, subcounty veterinary officers, and meat inspectors, and in workshops conducted with slaughterhouse workers in western Kenya. Dotted arrows indicate stakeholders with authority to introduce or enforce regulations; solid arrows indicate relationships between stakeholders. Blue boxes indicate a stakeholder whose influence could be leveraged to positively influence others. Further information about stakeholders can be found in Appendix 1.

From the focus group discussions, we identified stakeholders who play a role in AMR within the slaughterhouse context and how they relate to each other (Figure 2). Butchers emerged as prominent and powerful stakeholders who exerted pressure on others, sometimes impeding them from carrying out their work properly. For instance, a meat inspector stated, “Because you will find a carcass has already been prepared, you come and inspect you find the injection sites are all full of drugs, now when you tell this person this animal is supposed to be condemned, my friend you will be looking for trouble, you might even be forced to run.”

Participants recognized improper use of drugs as a factor driving resistance. Improper use included underdosing or overdosing animals and indiscriminate prescribing by professionals, such as medics and veterinarians. For example, a county veterinary officer stated, “If we vets also continue looking at all animals like they are ’antibiotic deficient,’ that is the disease we treat, this problem will continue escalating.”

Focus groups also identified challenges in dealing with bacterial AMR related to limited staff and inadequate funding, which led to underinvestments in infrastructure and equipment. Some slaughterhouses did not have access to reliable sources of clean water or lacked a perimeter fence, compromising biosecurity. Some slaughterhouse waste was disposed of indiscriminately, contaminating the surrounding environment. Participants cited the absence of surveillance to detect resistant pathogens and the lack of legislation requiring observance of withdrawal periods (periods during which animals are kept from the food chain while medications leave the body) on the farm as further hinderances.

Many participants noted that efforts to combat AMR required a multisectoral approach involving many stakeholders. In particular, they mentioned medics as a key group to include in public sensitization efforts because of the respect they command in the community.

In workshops for slaughterhouse workers, participants recognized they should seek medical advice before purchasing drugs, but admitted to often self-medicating or administering medications without a prescription because drugs, including some antibiotics, could be purchased directly from a chemist without prescription. The most popular antibiotic purchased for humans was amoxicillin and the most popular for livestock was oxytetracycline (Appendix 1).

Participants had experienced drug failure with antimalarial drugs in humans and with ectoparasitic and anthelmintic drugs in livestock. They understood that drugs often did not work because of misdiagnosis or misuse, including underdosing or not finishing the course. Participants provided various reasons for not finishing the course. A slaughterhouse worker in Bungoma said, “Sometimes you visit the chemist or clinic where a dose is prescribed for you but you don’t have enough cash so maybe the drugs cost like 600 and you have 200. So, because the doctor wants money, they tell you to go with a little drug and ask after how many days will you get the money? You tell him tomorrow. You take the drugs for a few days and notice a change then stop, which also contributes because you have not finished the dose.”

The workers understood that resistance was caused by a germ, and therefore transmitted in similar ways. They were also aware that their work increased their risk of infection because of their frequent contact with animals. A slaughterhouse worker in Busia said, “So, maybe there are diseases that affect the animals and is [sic] undergoing treatment. The animal is taken to the slaughterhouse without completing the treatment, when slaughtering the animal, there is the interaction between human and the animal. In case there are injury to the human, there may be mixing of blood of the animal and human blood and hence we can also be affected.”

Hygiene, both personal and at the workplace, was recognized as a key element in dealing with resistance. However, the uncertainty and poor pay associated with slaughterhouse work did not enable workers to purchase the required clothing and equipment. A slaughterhouse worker from Kakamega said, “...the pay you get is just enough to make ends meet but anything outside can’t be covered. The pay is too little for people who do hardy work in the slaughterhouse.”

Education was considered essential to understanding and mitigating the risk for bacterial AMR. For instance, a slaughterhouse worker in Busia stated, “Educate us on how we would be handling maybe the meat before it reaches [consumption]… Which other ways are we handling where, educating us on getting a knife, cutting meat, how to hang the meat so that it doesn’t get bacteria from the ground, like you said, dust usually contains bacteria, so you educate us before undertaking the work.”

## Conclusions

Our analysis identified butchers as powerful stakeholders; elsewhere butchers have been described as uncooperative and profit-driven (*9*,*10*). However, their influence could be leveraged to positively influence others, and they should be included in future education campaigns or intervention strategies. Limited finances were a recurring theme, leading to poor health-seeking behaviors despite interviewees being aware of recommended good practices. Many slaughterhouse workers understood how diseases are transmitted and the importance of hygiene measures, suggesting that engagement activities previously conducted in this area (*7*,*11*) or developed as part of this work (*12*) have been effective. However, translation of knowledge into practice is often hampered by the limited resources and external pressures that the workers face. 

In conclusion, long-term investments in slaughterhouses are needed to create an enabling environment that reduces the occupational risk to workers and safeguards the food they produce (*13*). Those investments need to be accompanied by investments in laboratory networks and surveillance efforts to better detect and mitigate AMR spread within the slaughterhouse context (*14*).

Appendix 1 Additional materials used in study of antimicrobial resistance in slaughterhouses, Kenya.

Appendix 2 Additional information about antimicrobial resistance in slaughterhouses, Kenya.
