# Supplementary material for: Antimicrobial Resistance in Slaughterhouses, Kenya
Source: Emerg Infect Dis. 2023 Oct;29(10):2155–8. doi: 10.3201/eid2910.230017 (PMC10521590; doi:10.3201/eid2910.230017)
Supplement: Appendix 1 — Additional materials used in study of antimicrobial resistance in slaughterhouses, Kenya. [file 23-0017-Techapp-s1.pdf]

Article DOI: https://doi.org/10.3201/eid2910.230017

EID cannot ensure accessibility for supplementary materials supplied by authors. Readers who have difficulty accessing supplementary content should contact the authors for assistance.

Antimicrobial Resistance in Slaughterhouses, Kenya

Appendix

Further Information about Stakeholders

Farmers are the primary source of animals and should ensure that animals sold for slaughter are healthy and not under treatment. Animals are bought by butchers, either directly or indirectly through traders, and taken for slaughter. Butchers were considered powerful stakeholders because they often had political connections, and they sometimes impeded other stakeholders, including meat inspectors, from executing their work properly. Slaughterhouses are either owned by private individuals or by the county government, who are responsible for procuring equipment and maintaining the slaughterhouse environment. Before the slaughterhouse is constructed, the National Environment Management Authority performs an environmental assessment of the site, after which they monitor the slaughterhouse building environment and waste disposal process. Meat inspectors should be present in all slaughterhouses. They ensure that slaughterhouse workers have been trained, are licensed, and are wearing clean and adequate clothing. They conduct antemortem and postmortem inspections and condemn unsafe meat. They inspect the meat box used to transport meat from the slaughterhouse to the butchery before issuing certificates of transport. Public health inspectors from the Ministry of Health check the cleanliness of butcheries and issue medical certificates for all slaughterhouse workers and meat carriers. Consumers could drive change by choosing to buy inspected meat from clean butcheries. Livestock antibiotics can be purchased from veterinary drug pharmacies (agrovets). The Veterinary Medicine Directorate ensures that drugs are sold on

prescription and that the withdrawal period is respected. A multisectoral approach that involved medics was considered desirable when dealing with antimicrobial resistance.

Data Collection Tool for Focus Group Discussions with Sub-County Veterinary Officers

Thank you for agreeing to participate in this meeting. Through the following questions we hope to better understand issues related to animal welfare, antimicrobial resistance, public health and food hygiene in the slaughterhouse context. There are no right and wrong answers, so please feel free to express your thoughts and opinions.

We shall first start with a couple of more general questions.

[Governance]

1. Which pieces of legislation govern your work?

1a. Of these, are there any County-specific legislations?

1b. If yes, were you involved in their development?

1c. Of the legislations that you've mentioned, which are specifically related to slaughterhouse activities (e.g., welfare at slaughter, meat inspection)?

1d. Are you able to implement these legislations?

1e. In your opinion, are these legislations sufficient to carry out your work?

1f. How would you compare your experience in implementing these legislations before and after devolution?

[Challenges]

2. Besides legislation issues mentioned above, what other challenges do you encounter in your work?

We're now going to ask you a set of questions related to the following sub-topics: Animal welfare, Antimicrobial resistance, Public Health and Food Hygiene.

Let's start with Animal Welfare:

[Awareness]

3. Are you aware of *Animal Welfare* within your work context?

[Activities]

4. What activities related to *Animal Welfare* do you carry out during your work?

[Actors involved]

5. Who do you interact with when implementing *Animal Welfare* activities during your work?

[Concerns/Challenges]

6. What are your main concerns/challenges when trying to implement *Animal Welfare* during your work activities?

7. How do you think *Animal Welfare* will change in 10 years' time?

8. How would YOU want *Animal Welfare* to change in 10 years' time? *("If you were king for a day, what would you change?")*

Thank you for that. Now we shall move on to issues related to Antimicrobial resistance.

[Awareness]

9. Are you aware of *Antimicrobial resistance* within your work context?

[Activities]

10. What activities related to *Antimicrobial resistance* do you carry out during your work?

[Actors involved]

11. Who do you interact with when implementing *Antimicrobial resistance* activities during your work?

[Concerns/Challenges]

12. What are your main concerns/challenges when trying to implement *Antimicrobial resistance* concepts during your work activities?

13. How do you think *Antimicrobial resistance* will change in 10 years' time?

14. How would YOU want *Antimicrobial resistance* to change in 10 years' time? *("If you were king for a day, what would you change?")*

Now, for the last set of questions we'll focus on public health and food hygiene.

[Awareness]

15. Are you aware of *Public health and Food hygiene* within your work context?

[Activities]

16. What activities related to *Public health and Food hygiene* do you carry out during your work activities?

[Actors involved]

17. Who do you interact with when implementing *Public Health and Food Hygiene* activities during your work?

[Concerns/Challenges]

18. What are your main concerns/challenges when trying to implement *Public Health and Food Hygiene* during your work activities?

19. How do you think *Public Health and Food Hygiene* will change in 10 years' time?

20. How would YOU want *Public Health and Food Hygiene* to change in 10 years' time? *("If you were king for a day, what would you change?")*

We've now almost reached the end of the questions. Before ending…

21. Could you EACH please rank the following challenges (list put together during discussion), starting with those you think should be dealt with first? We can then discuss them together after.

(*hand out piece of paper to each participant so they can write things down).

22. Of the three themes we've discussed today (i.e., animal welfare, antimicrobial resistance, and public health and food hygiene), which one do you think would be easiest to address?

Before ending, could we kindly ask you to write on a piece of paper what you found most and least useful about this meeting? Thank you.

(*provide them with a piece of paper to write).

Thank you for your time. Do you have any other thoughts or comments you would like to share with us?

Thank you.

Questions for Slaughterhouse worker workshops:

a) Animal Welfare

- What do you think this is?
- What do you do about it? **strengths*
- What can be done in your workplace? **opportunities*
- What keeps you from making these changes? **weaknesses/threats*
- Any other challenges?
- How do you like to receive information?

b) Antimicrobial resistance

- What do you think this is?
- What do you do about it? **strengths*
- What can be done in your workplace? **opportunities*
- What keeps you from making these changes? **weaknesses/threats*
- Any other challenges?
- How do you like to receive information?

c) Public health and food hygiene

- What do you think this is?
- What do you do about it? **strengths*
- What can be done in your workplace? **opportunities*
- What keeps you from making these changes? **weaknesses/threats*
- Any other challenges?

- How do you like to receive information?

d) Human welfare and worker satisfaction

- Where did you learn how to do your work?
- How do you feel about your work?
- How do you get paid?
- What are the challenges you face?
- How do you like to receive information?

Appendix 1 Table. The drugs used in humans and for livestock, and the reasons given for taking a particular drug, as mentioned by slaughterhouse workers in western Kenya during workshops on antimicrobial resistance in the slaughterhouse context.

Drug Class	Drug	Product Name	Reason for Use
Humans			
Antibiotic	Amoxicillin	NS	Throat pain; Cough
	Ciprofloxacin	Ciproflax	Cough; Cold
	Ethambutol	Actol	Diarrhea
	Neomycin, Bacitracin & Polymyxin	Grabacin	Wounds
	Trimethoprim/Sulfamethoxazole	Septrin	Cough; Cold
Anthelmintic	NS	NS	Worms
Anti-histamine	Celestamine	NS	Cold
	Ceptrizine	NS	Cold; Cough
	Chlorphenamine maleate	Piriton	Lack of appetite; Cough; Insomnia; Cold
Anti-inflammation	Aspirin	Mara Moja	Weakness; Malaria; Strong cough
	Diclofenac	NS	Fever; Toothache
	Ibuprofen	Brufen	Cough; Body pains
	Indomethacin	Indocid	
	Paracetamol	Panadol	Headache, Stomach ache; Body pains; Fever
		Sona Moja	Headache; Dizziness
		Hedex	Headache; Throat pain; Weakness
Anti-malarial	Artemisinin-Based Combination Therapy	NS	Malaria
	Artemether-Lumefantrine	NS	Malaria
	Metakelfin	NS	Fever; Headache; Joint pains
Anti-protozoal	Metronidazole	Flagyl	Diarrhea; Stomach upsets
Combination medicine	Chlorpheniramine maleate, Dextromethorphan, Hydrobromide, Paracetamol & Phenylephrine	Flugone	Cough
	Chlorpheniramine maleate & Phenylpropanolamine hydrochloride	Coldcap	Cold
	Glycyrrhiza extract, Menthol, Oil of Anise, Oil of Peppermint, Tincture of Capsicum, Oil of Pine, Oil of Eucalyptus & Creosote	Cofta	Throat pain
Multivitamin	B-complex minerals	Action	Headache
Oral Rehydration Solutions	NS	NS	Diarrhea
Traditional medicine	Aloe vera	NA	Malaria; Pain; Foot wounds
	Eggs & Soda ash	NA	Cough
	Herbs	NA	Worms
	Lemon, Garlic & Ginger	NA	Cough
	Neem	NA	Skin rashes
Livestock			
Antibiotic	Oxytetracycline hydrochloride	Egocin	Chicken disease; Lack of appetite
	Tetracycline	Tetrax	Swollen abdomen
Anthelmintic	Albendazole	Albafas	NS
	Levamisole	Nilzan	NS
Ectoparasiticide	Amitraz	Triatix	NS
	Chlorpyrifos & Cypermethrin	DuoDip	NS
Hormones	Oxytocin	Vetox	Chicken disease
Multivitamin	Vitamin B12 & Phosphorus	Catasol	Increase appetite
Traditional medicine	Herbs	NA	Foot problems
	NS	NA	Diarrhea

*NA, not applicable; NS, not specified.
